# Supportive periodontal therapy: individual patients’ perception of various professional interventions

**DOI:** 10.1186/s12903-026-07656-5

**Published:** 2026-01-16

**Authors:** Miriam Cyris, Leah Kopetzki, Dominik M. Schulte, Christoph E. Dörfer, Johannes C. Ehrenthal, Christian Graetz

**Affiliations:** 1 Clinic of Conservative Dentistry and Periodontology, University Hospital Schleswig- Holstein, Campus Kiel, Arnold-Heller-Str. 3, Haus B, Kiel, 24105 Germany; 2 Department of Internal Medicine I, Division of Endocrinology, Diabetes and Clinical Nutrition, University Hospital Schleswig-Holstein, Kiel, Germany; 3 Department of Psychology, University of Cologne, Cologne, Germany

**Keywords:** Heart rate, Dental fear, Compliance, Periodontitis, Supportive periodontal therapy, Tooth loss

## Abstract

**Background:**

Evidence on psychophysiological responses such as heart rate (HR) and electrodermal response (EDR) during long-term supportive periodontal therapy (SPT) is limited. This observational cross-sectional pilot study aimed to explore associations between patient-related factors and physiological stress markers during routine SPT visits, focusing on adherence-related variables.

**Methods:**

A sample of *n* = 75 patients was examined in a questionnaire-based, cross-sectional survey, indicating sufficient adherence to SPT of ≥ 2 years (a maximum deviation ± 6 months between SPT intervals) at a specialized department for periodontology. At the preliminary last SPT visit, in addition to dental parameters, we assessed socio-demographic, treatment-related (critical attitudes/complaints), psychological variables—such as dental fear, oral health-related quality of life, dental anticipatory trauma symptoms, childhood traumata, depression, and personality functioning—and vital parameters such as HR and EDR. The primary endpoint was the difference in HR and EDR across predefined procedural clusters reflecting varying invasiveness (three clusters: non-invasive (n-iC), minimally invasive (m-iC), and invasive (iC). Analyses were performed using non-parametric tests and exploratory correlation analyses.

**Results:**

The mean (standard deviation) of HR differed significantly across intervention clusters (n-iC/m-iC/iC: 73.06[11.96]/67.66[9.60]/69.46[10.00] bpm; *p* ≤ 0.001), while EDR revealed no significant differences (n-iC/m-iC/iC: 69.64[156.65]/61.72[172.20]/75.10[154.07] µS; *p* ≥ 0.075). EDR negatively correlated with the number of teeth m-iC-interventions (*r* = -0.423; *p* = 0.003). Moreover, EDR and HR were significantly related to certain variables such as long-term medication and number of general diseases (*p* < 0.001). Significant correlations included a positive association between Oral Health Impact Profile scores and dental anxiety (*r* = 0.233; *p* = 0.040) and a negative correlation with the total number of teeth (*r* = -0.378; *p* < 0.001).

**Conclusion:**

Socio-demographic and treatment-related factors were intertwined with patient and dental parameters in SPT and appeared to influence adherence. HR varied across clusters, but no clear link between vital signs and psychological stress could be established.

**Trial registration:**

The clinical trial was prospectively registered in the DRKS—German Clinical Trials Register (https://www.drks.de) with the registration-ID DRKS00031969 (06/05/2023).

**Supplementary Information:**

The online version contains supplementary material available at 10.1186/s12903-026-07656-5.

## Background

Periodontitis is a chronic, biofilm-associated inflammatory disease of the periodontium, causing tooth loss if untreated [1–3]. More than 65% of the global population suffers from it [4]. Periodontitis therapy aims to eliminate inflammation, stop progression, and, if possible, regenerate lost tissue. Long term success relies on regular supportive periodontal therapy (SPT) [5–7]. However, SPT compliance and adherence are often unsatisfactory, so it is crucial to investigate factors affecting this [8].

Dental anxiety (DA) [9–11] and patient perception, including discomfort and pain, significantly affect compliance [12]. Other factors are a lack of information, socioeconomic status, involvement in decision making, self-destructive behavior, economic factors, stressful life events, and perceived indifference of the dentist [13, 14]. Nearly 10% of participants in a study by Humphris et al. [15] reported severe DA, often due to past negative experiences [16, 17]. Anxious patients are 2.3 times more likely to perceive dental treatment as painful [18]. Periodontitis patients generally have higher DA [19], but it decreases during periodontal therapy [20]. DA is associated with several health-related consequences, including poor oral health [21–24], poor oral health-related quality of life (OHRQoL) [25–27], and avoidance of future dental treatment [28, 29] as well as negative impacts on the clinical outcomes of active periodontal therapy (APT) [30] and SPT [31].

Dentists should develop strategies to alleviate patients’ fears and make treatments painless to improve their compliance and adherence to APT and SPT, which will benefit their long-term success [32]. However, there is little data on which SPT interventions cause discomfort. Additionally, several questionnaire-based studies have investigated various factors for compliance with and adherence to SPT [31, 33] but never in combination with vital parameters measured in real time. Studies should include questionnaires and vital parameters such as heart rate (HR) and electrodermal response (EDR), which could act as markers for stress, discomfort, or pain [34].

This study investigates stress and discomfort during specific SPT interventions (primary parameter: HR) to identify methods to reduce patient stress and pain, thereby increasing their compliance with and adherence to better periodontal maintenance.

## Materials and methods

### Sample

For this observational cross-sectional pilot study, we recruited patients (*n* = 79) undergoing SPT at a university hospital in Kiel, Germany, irrespective of SPT duration. These participants were adherent to SPT at patient-specific intervals (3–12 months frequency) and had attended previous SPT sessions in our department after prior periodontitis diagnosis. Four patients declined participation, leaving 75 patients who provided written consent before the study began. The study was designed as single-visit observational assessment in which all data were collected at one point between 3 July and 10 August 2023 and included no longitudinal follow-up.

Participants were classified according to the periodontal classification from 2018 [35] based on the day of the SPT visit using the patient’s most recent clinical findings. Success of periodontal treatment (APT and SPT) was assessed following the Swiss quality guidelines for periodontology [36] and those of Feres et al. [37].

Due to the lack of comparable studies in adult dental populations or periodontology, this study was planned as a pilot study. Nevertheless, to support the sample size planning, we referred to an investigation in pediatric dentistry [38], which indicated that a sample size of *n* = 51 participants would be sufficient to detect small differences (less than 5%) in HR as a primary outcome measure between procedural conditions, assuming 80% power. While not directly transferable, this approximation served as a pragmatic reference point for the pilot design.

**Fig. 1:**
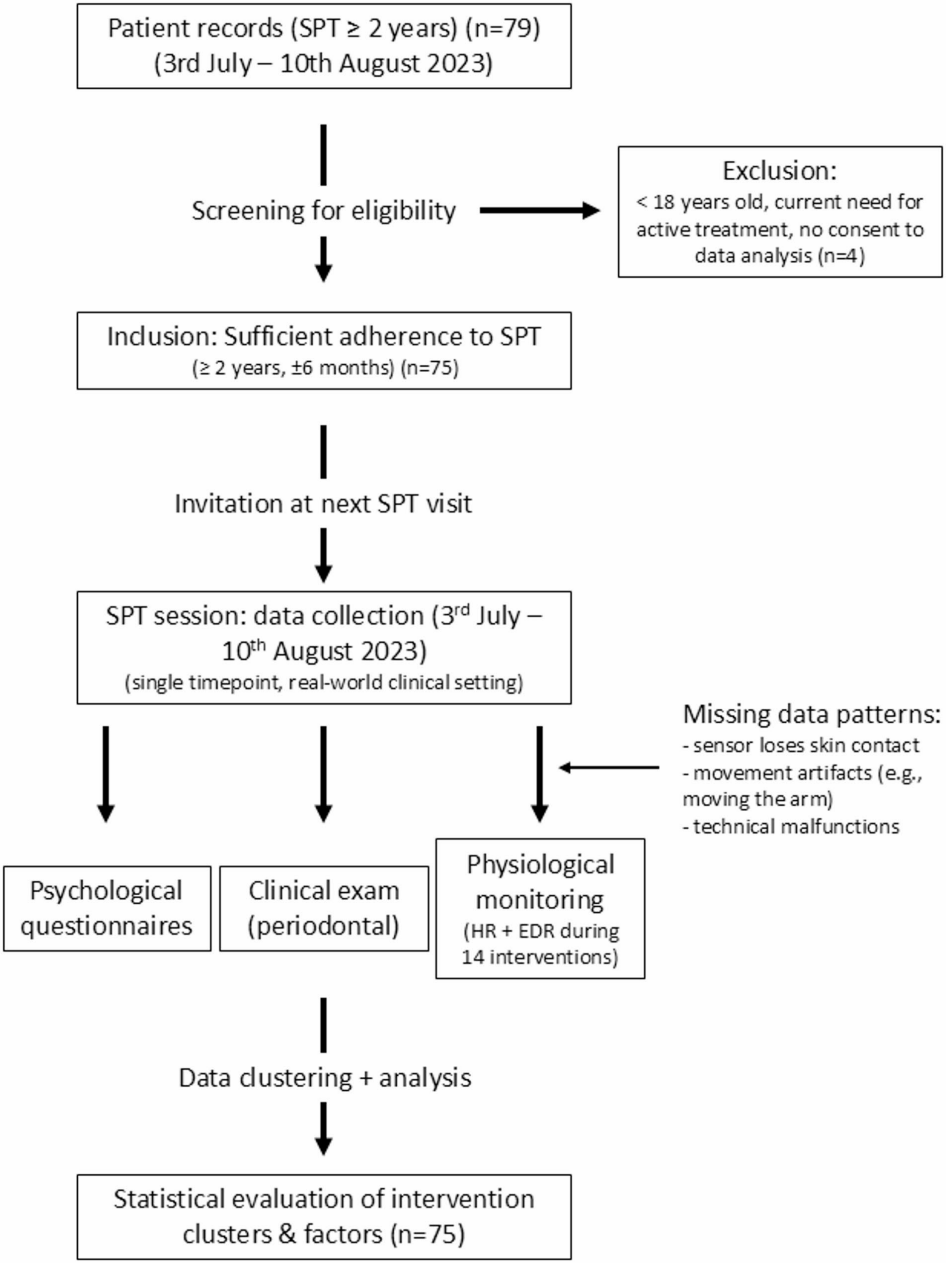
Schematic illustration of the study design. Patient flow (n=79 approached, n=75 included) and sources of missing data are shown alongside the structure of the data collection during the SPT session. Legend: Patients were selected based on ≥ 2 years of documented adherence to supportive periodontal therapy (SPT). At the subsequent SPT, session, psychological, clinical, and physiological data were collected. Physiological signals (HR, EDA) were recorded continuously during 14 defined interventions. Interventions were grouped into clusters by invasiveness level and analyzed accordingly

A detailed flow diagram illustrating the study design is provided in Fig. 1. The diagram also summarizes missing-data patterns and reasons for exclusion.

### Supportive periodontal therapy

As a maintenance phase, SPT follows successful APT with non-surgical periodontal therapy (NSPT), sometimes flanked by additional surgical interventions or adjuvant antibiotics therapy [7]. Despite its necessity for life, SPT cost coverage in Germany’s health insurance system has been limited to two years since July 2021, according to the regulations of the Gemeinsamer Bundesausschuss (G-BA). SPT intervals (4–10 months) depend on the individual rate of progression of periodontitis (grades A, B, or C based on the current classification [39]). G-BA recommendations for SPT include an oral hygiene check; oral hygiene instruction, if necessary; professional mechanical plaque removal; periodontal status assessment, for example, probing depths (PD), gingival recession (GR), bleeding on probing (BOP), tooth mobility (TM), furcation involvement (FI); and subgingival instrumentation at sites with PD ≥ 5 mm and/or PD ≥ 4 mm with BOP.

### Outcomes

#### Vital parameters

In this study, vital parameters such as HR and EDR were non-invasively and simultaneously measured during SPT interventions. HR, linked to the autonomic nervous system, helps estimate stress levels [40], while EDR indicates an increase in sympathetic tone [41]. These two measurements were continuously recorded by an HR monitor on the patient’s wrist during the SPT visit. With a focus on usability and reducing burden of more complex measurement systems for this pilot study, the CARDIOWATCH 287-2 (Corsano Health B.V., AN The Hague, The Netherlands [abbreviation: device1]) [42] and the EmbracePlus wristband (Empatica Inc., Cambridge MA, United States [abbreviation: device2]) [43] were used for continuous HR and EDR. Both devices use photoplethysmography (PPG sensors) to measure HR. Regarding EDR, device 1 has two electrodes at the underside of its bracelet, recording both tonic and phasic EDR, with a sampling rate of 25 Hz. Device 2 continuously measures the tonic level of EDR, referred to as the skin conductance level (SCL), via two electrodes positioned on the inner wrist, at a sampling rate of 4 Hz. Each participant was measured using one device only, depending on system availability. No simultaneous dual-device recordings were performed. Monitoring and documentation of individual interventions and measurements of vital parameters during SPT was conducted by one investigator (L.K.). The following interventions were recorded: (1) welcome interview, (2) extraoral/intraoral examination, (3) measurement of periodontal parameters (e.g., PD, GR, BOP, TM, FI), (4) local anesthesia, (5) instrumentation with curettes (American Eagle Instruments, Missoula, MT, USA), (6) instrumentation with sonic scaler (Proxeo, W&H, Bürmoos, Austria), (7) instrumentation with ultrasonic scaler (Prophylaxis Master, E.M.S. Electro Medical Systems S.A., Nyon, Switzerland), (8) instrumentation with airflow (Prophylaxis Master, E.M.S. Electro Medical Systems S.A., Nyon, Switzerland), (9) instrumentation with rotating rubber cup (Prophy-Cups, KerrHawe SA, Bioggio, Switzerland) and polishing paste (ProphyCare Prophy-Paste CCS Polishing Paste medium, RDA 170), (10) use of oral hygiene aids (toothbrush, interdental brush, dental floss, etc.), (11) instrumentation with rotating brushes interdental (CURADEN GmbH, Stutensee, Germany), (12) fluoridation/subgingival application of medication (e.g., chlorhexidine gel), (13) demonstration and interaction with the patients for oral hygiene instruction/motivation or answering questions and, if occurring, (14) treatment breaks (e.g., patient waits for dentist). For statistical analysis, these 14 interventions were classified into intervention clusters as non-invasive (n-iC [*n* = 5]: 1, 2, 12, 13, 14), minimally invasive (m-iC [*n* = 5]: 3, 8, 9, 10, 11), and invasive (iC [*n* = 4]: 4, 5, 6, 7). Interventions were clustered based on physical invasiveness and expected psychophysiological activation, with non-invasive procedures defined as those involving minimal or no physical manipulation (e.g., fluoridation, subgingival medication, patient communication). A detailed mapping of each intervention to its respective cluster is provided in Table S1. Because access to raw signal streams was limited by proprietary firmware, data preprocessing (e.g., artifact detection, filtering, or synchronization corrections) could not be manually customized. Instead, manufacturer-embedded filtering was applied automatically, and temporal segmentation of the physiological data was aligned post-hoc to the documented treatment phases. No separate baseline or resting phase was implemented before data collection, as the study aimed to capture real-world psychophysiological responses during routine SPT.

#### Local anesthesia

Local anesthesia (1.7 mL of Sopira^®^ 1:200,000 with epinephrine) was administered in only four of seventy-five patients (5.3%) directly after the welcome phase and before instrumentation. Given this low incidence and timing prior to physiological data collection, we considered its effect on HR or EDR negligible; nonetheless, it is acknowledged as a potential confounder.

#### Questionnaires

Directly prior to the SPT visit, all participants answered the following questionnaires.

#### Dental fear

The Modified Dental Anxiety Scale (MDAS) was used to assess DA, regardless of the general level of anxiety [15]. The MDAS consists of five items, each with five answers ranging from “not anxious” to “extremely anxious”; it is the extended version of Corah’s Dental Anxiety Scale.

#### Oral health-related quality of life

A 14-item version of the Oral Health Impact Profile (OHIP-14) was used in this study, which demonstrated good reliability and validity [44]. The items were answered on a 5-point Likert scale from 1 = “never” to 5 = “very often.”

#### Dental anticipatory trauma symptoms

We used six items of the Impact of Event Scale-revised (IES-R) IES-6 adapted to the dental situation to identify post-traumatic stress reactions and stress disorders in test subjects. IES-6 appears to be a robust brief measurement of post-traumatic stress reactions [45].

#### Childhood traumata

The Childhood Trauma Screener (CTS) also was used in this study. The CTS is a questionnaire with five items asking about the frequency of sexual, emotional, and physical abuse as well as emotional and physical neglect [46]. This brief instrument captures key forms of adverse childhood experiences (ACEs), which have been widely linked to long-term health outcomes, as shown in the foundational ACE studies by Felitti, Anda [47].

#### Personality functioning

The personality functioning level, which depicts a dimensional assessment of personality disorders, was determined using the 12-item short version of the Operationalized Psychodynamic Diagnosis Structure Questionnaire (OPD-SQS), which proved to be a viable screening tool in comparable studies [48, 49].

#### Depression and anxiety

Overall psychological burden, which can be measured by levels of depression and anxiety, was assessed using the Patient Health Questionnaire 4 (PHQ-4). This 4-item version is an ultra-short questionnaire consisting of a 2-item depression scale (PHQ-2) and a 2-item anxiety scale (GAD-2) with good reliability and validity [50].

### Other variables

#### Independent variables

For this study we additionally recorded year of birth, age, and gender of the patients. We also identified smokers, the general number of somatic diseases, patients taking long-term medication, and subjects with diabetes mellitus, cardiovascular diseases, and oncological conditions. As mentioned previously, all patients were classified according to the current classification of periodontal diseases [35]. Treatment goals achieved in SPT were categorized to the treatment target according to the Swiss quality guidelines for periodontology [36] (A+: no pockets > 4 mm with frequent BOP, no visible (mineralized) biofilm, non-smoker, or successfully quit smoking; A: no pockets > 4 mm with regularly BOP, no probing values > 5 mm, only a few sites with visible (mineralized) biofilm; B: individual pockets > 4 mm with regularly BOP, clinical attachment loss (CAL) and PD not stable everywhere, several areas with visible (mineralized) biofilm; C: generalized BOP, progressive significant CAL at several sites), and to the guidelines presented by Feres et al. [37] (dichotomized for treatment success of at ≤ 4 sites with PD ≥ 5 mm or ≤ 10% of sites with BOP).

Moreover, on the patients’ level, the total number of teeth and the number of molars, premolars, and incisors were recorded. For periodontal findings, the number of sites with PD ≤ 3 mm, PD = 4 mm, with PD ≥ 5 mm and with PD = 4 mm with BOP and ≥ 5 mm with or without BOP, as well as the mean value of PD were documented. Additionally, the mean value of CAL was calculated as the sum of PD and GR. The records also consisted of the number of teeth with FI according to Hamp, Nyman [51] of degree 0/1/2/3 and the number of teeth with TM according to Lindhe and Nyman [52] of degree 0/1/2/3. BOP and plaque control recorded (PCR) according to O’Leary, Drake [53] were calculated in the percentage of all teeth per patient.

#### Data management and statistical analysis

Informed consent was obtained from all patients for the analysis of their data documented during periodontal therapy. Data were sampled in an internal database of the Department of Periodontology, Kiel, Germany (PAROPS-DB 2022 V 1.0.0, Leipzig, Germany). Descriptive analyses were conducted. In addition to using the t-tests to assess possible differences in physiological variables for HR and EDR, we calculated the effect sizes for samples with paired values (Hedges’ g) to quantify the size of those differences. Analysis of variance (ANOVA) was used to identify significant differences considering HR and EDR. Due to logistical constraints during the study period, each participant was monitored using only one of the two available devices (either device 1 or device 2). As no participant was assessed with both devices in parallel, a direct comparison or agreement analysis (e.g., Bland–Altman) between devices was not applicable. Accordingly, device-related differences were explored using the Mann–Whitney U test of independent samples. To compare HR and EDR throughout the intervention clusters for both device1 and device2, the Kruskal–Wallis test of independent samples was applied. Pearson correlation was used to assess associations between questionnaire variables (e.g. MDAS) and physiological measures. All analyses were conducted with IBM SPSS 27 (SPSS, Chicago, IL, USA). For transparency, exact p-values and standardized within-subject effect sizes (Cohen’s dz where applicable) are reported for key contrasts, together with descriptive statistics and 95% CIs.

## Results

### Demographic and periodontal data

The demographic data and periodontal behavior of the 75 included subjects are presented in Table 1.

**Table 1:** Demographic and periodontal data

	**Number of patients (percent)**
Sex (female/male)	49 (65.3)/26 (34.7)
Smoking (no/yes)	61 (81.3)/14 (18.7)
Number of general disease (0/1/2/≥3)	31 (41.3)/29 (38.7)/12 (16)/3 (4.0)
Coronary heart disease (no/yes)	43 (57.3)/32 (42.7)
Diabetes mellitus (no/yes)	66 (88.0)/9 (12.0)
Cancer or condition after cancer (no/yes)	68 (90.7)/7 (9.3)
long-term medication intake (no/yes)	28 (37.3)/47 (62.7)
Periodontitis stage	
I	3 (4.0)
II	13 (17.3)
III	33 (44.0)
IV	26(34.7)
Periodontitis grade	
A	0 (0)
B	44 (58.7)
C	31 (41.3)
Localized/generalized periodontitis	36 (48.0)/39 (52.0)
Feres criteria fulfilled (no/yes)	55 (73.3)/20 (26.7)
Swiss quality guidelines for periodontology (A+/A/B/C)	1 (1.3)/8 (10.7)/59 (78.7)/7 (9.3)
	**Mean ± SD (range)**
Age of subjects (in years)	65.3 ± 12.4 (34–87)
SPT appointment time (in minutes)	41.5 ± 13.7 (13.2–80.0)

*SPT* Supportive Periodontal Therapy

Apart from the dental data seen in Table 2, we calculated a maximum of exposed root surface per subject of 1.84 (2.44) mm at *n* = 23.55 (39.66) sites per patient.

**Table 2:** Dental parameters

	Mean ± SD (range)
**Number of teeth per patient**	
total	22.5 ± 5.7 (2–29)
molars	5.5 ± 2.2 (0–10)
premolars	6.3 ± 1.9 (1–8)
incisors	10.7 ± 2.5 (1–12)
**Number of molars with FI per patient**	
grade 0	6.4 ± 3.1 (0–12)
grade I	0.5 ± 1.2 (0–5)
grade II	0.1 ± 0.4 (0–2)
grade III	0.2 ± 0.6 (0–3)
**Number of teeth with TM per patient**	
grade 0	22.2 ± 6.2 (0–30)
grade I	0.5 ± 2.5 (0–20)
grade II	0 ± 0.1 (0–1)
grade III	0 ± 0.1 (0–1)
**CAL (in mm)**	3.3 ± 0.9 (2–6)
**PD (in mm)**	2.8 ± 0.4 (2–4)
**Number of sites with PD per patient**	
0–3 mm	117 ± 32.1 (7–166)
4 mm	12.8 ± 9.3 (1–53)
≥ 5 mm	6.5 ± 7.2 (0–36)
4 mm with BOP and ≥ 5 mm with or without BOP	10.4 ± 8.8 (0–36)
**BOP per patient (in percent)**	13.4 ± 19.4 (0–100)
**PCR per patient (in percent)**	29.4 ± 19.0 (0–100)

*CAL* Clinical Attachment loss (in mm), *PD* Probing Depths (in mm), *BOP* Bleeding on Probing (in percent), *PCR* Plaque Control Record (in percent)

### Psychological data

Regarding the questionnaires, the OHIP-14 indicated no restriction in 28.0% of the patients (*n* = 21, 0 points), minor restriction in 41.3% (*n* = 31, 1–4 points), moderate restriction in 20.0% (*n* = 15, 5–11 points), and severe restriction in 10.7% (*n* = 8, ≥ 12 points) in oral health-related quality of life. The MDAS score suggested that 12 subjects of this study did not have DA (16%, ≤ 5 points), 42 had mild DA (56%, 6–10 points), 19 suffered from moderate DA (25.3%, 11–18 points), and 2 patients exhibited severe DA (2.7%, ≥ 19 points).

The OHIP-14 positively correlated with most of the other psychological variables: the MDAS (*r* = 0.233; *p* = 0.040), the IES-6 (*r* = 0.424; *p* < 0.001), the OPD-SQS (*r* = 0.308; *p* = 0.005), and the PHQ-4 (*r* = 0.280; *p* = 0.011). This demonstrates that patients with poor OHRQoL usually also have higher levels of DA, symptoms of dental-related post-traumatic stress disorder (PTSD), personality disorders, and depression and anxiety. Furthermore, the MDAS positively related to the IES-6 (*r* = 0.333; *p* = 0.002) and the OPD-SQS (*r* = 0.222; *p* = 0.047); therefore, patients with higher levels of dental anxiety often exhibit more symptoms of dental-health related PTSD and general personality disorders as well. The MDAS (*r* = −0.243; *p* = 0.036) and the CTS (*r* = −0.242; *p* = 0.043) indicated a negative association with PCR, meaning that subjects with DA or childhood trauma exhibited lower plaque rates. However, the OHIP-14 negatively correlated with the number of teeth (*r* = −0.378; *p* < 0.001), and a low OHRQoL was often found together with fewer teeth.

The periodontal stage positively correlated with OHIP-14 (*r* = 0.271; *p* = 0.012) and IES-6 (*r* = 0.236; *p* = 0.030), and the grade corresponded with MDAS (*r* = 0.285; *p* = 0.008), OHIP-14 (*r* = 0.314; *p* = 0.003), and IES-6 (*r* = 0.302; *p* = 0.005). The periodontal extent positively related only to OHIP-14 scores (*r* = 0.229; *p* = 0.035). This indicates that the more severe the stage, grade, and/or extent of periodontitis, the worse the OHRQoL. A higher stage usually also comes with more PTSD-related burden. The same is true for a higher grade and it is associated with greater DA levels.

A negative relationship was found between patients’ age and MDAS (*r* = −0.289; *p* = 0.007), OHIP-14 (*r* = −0.217; *p* = 0.046), and IES-6 (*r* = −0.285; *p* = 0.008). This means that younger patients were more likely to suffer from higher levels of DA and symptoms of dental-related PTSD.

### Physiological data

The mean HR differed significantly across the intervention clusters (n-iC and m-iC [*p* < 0.001], n-iC and iC [*p* = 0.002], and m-iC and iC [*p* = 0.003]). N-iC had the highest HR and m-iC the lowest: During n-iC, the mean (SD) HR was 73.06 bpm (11.96); for m-iC, it was 67.66 bpm (9.60); and the mean (SD) HR of iC indicates a value of 69.46 bpm (10.00). For EDR there were no significant differences (n-iC and m-iC [*p* = 0.075], n-iC and iC [*p* = 0.284], and m-iC and iC [*p* = 0.886]), The mean (SD) of 69.64µS (156.65) for n-iC and 61.72µS (172.20) for m-iC. Additionally, the iC has a mean (SD) EDR of 75.10µS (154.07). No significant correlation was found between mean HR and mean EDR (*r* = −0.099; *p* = 0.428).

The number of teeth was negatively associated with EDR m-iC (*r* = −0.423; *p* = 0.003): fewer teeth had higher EDR figures in m-iC. Prosthetic restoration, BOP, PCR, patient’s age, periodontal stage, grade and extent, Feres criteria, and Swiss quality guidelines did not significantly affect the mean HR and mean EDR. HR and EDR were significantly related to certain variables such as long-term medication and number of general diseases as well as by the measuring device (*p* < 0.001).

### Measuring devices

Each participant was recorded with only one of the two devices (Cardiowatch 287-2 or EmbracePlus), depending on availability. No simultaneous dual-device measurement occurred; therefore, comparisons between devices represent independent groups rather than paired data. The two groups with diverging measuring devices, device1 and device2, differed significantly in mean HR (*p* = 0.045), mean EDR (*p* < 0.001), minimum EDR (*p* < 0.001), and maximum EDR (*p* < 0.001). Our study reveals that in both measuring devices there were significant differences in the mean HR between m-iC and n-iC (device1: *p* = 0.005; device2: *p* < 0.001), but they were not significant for the other intervention clusters. For the mean EDR, both wristbands did not demonstrate any significance in statistical tests on all of the intervention cluster pairings (*p* > 0.005).

For device2 group, we found that only for the minimal HR was the distribution of the three intervention clusters identical (*p* = 0.111); this was not the case for the mean HR (*p* = 0.008) and the maximum HR (*p* = 0.002). For EDR, the mean (*p* = 0.521), minimal (*p* = 0.901), and maximal values (*p* = 0.265) for n-iC, m-iC, and iC were identical. Regarding device1 group, the mean (*p* < 0.001), minimal (*p* = 0.002), and maximal HR (*p* < 0.001) differed between intervention clusters. This was not the case for the mean (*p* = 0.556), minimal (*p* = 0.592), and maximal EDR (*p* = 0.681), as these were identical across n-iC, m-iC, and iC. This means that, from our findings, device1 seems to be able to measure HR quite well but not EDR. Device2 generated less valid data for both vital parameters.

### Associations and correlations between psychological and physiological data

As Table 3 indicates, there is no significant correlation between the MDAS and both measured physiological vital parameters, HR and EDR. Moreover, there was no significant association between the OHIP-14, the IES-6, the OPD-SQS12, the PHQ-4, and the CTS with HR and EDR, with the exception that PHQ-4 has a negative correlation with HR m-iC (*r* = −0.269; *p* = 0.047), meaning the more the patients suffer from depression and anxiety, the lower the HR in m-iC.

**Table 3:** Results of the correlation between the modified dental anxiety scale and heart rate/electrodermal response

Variable x	Variable y	*r*, *p*	Pearson correlation
MDAS	overall mean HR	*r* = 0.113; *p* = 0.311	none
MDAS	n-iC mean HR	*r* = 0.156, *p* = 0.159	none
MDAS	m-iC mean HR	*r* = 0.063, *p* = 0.642	none
MDAS	iC mean HR	*r* = 0.196, *p* = 0.104	none
MDAS	overall mean EDR	*r* = 0.077, *p* = 0.538	none
MDAS	n-iC mean EDR	*r* = 0.034, *p* = 0.787	none
MDAS	m-iC mean EDR	*r* = 0.216, *p* = 0.140	none
MDAS	iC mean EDR	*r* = 0.079, *p* = 0.551	none

*MDAS* Modified Dental Anxiety Scale, *HR* Heart Rate (beats per minute (bpm)), *n-iC* Non-Invasive Intervention Cluster, *m-iC* Minimally Invasive Intervention Cluster, *iC* Invasive Intervention Cluster, *EDR* Electrodermal Response (µS)

Summary statistics for additional questionnaire data not shown in the manuscript can be made available upon request.

## Discussion

The aim of this observational, cross-sectional pilot study was to research which SPT interventions are associated higher stress levels in patients with the goal of improving patients’ compliance and adherence for better periodontal maintenance results, given its relevance after successful active periodontal treatment [5–7]. As studies with psychophysiological data in dental patients is lacking, the intention was not to test pre-defined causal relationships but to generate hypotheses and feasibility data for future controlled studies. Taken together, our pilot study suggests that while heart rate varied across procedural clusters, no consistent association with psychological stress levels was found. Some clinical and psychological parameters—such as DA and multimorbidity—showed plausible but exploratory links to physiological data. These findings support our assumption that stress responses in SPT are shaped by overlapping patient- and treatment-related factors.

Our sample comprises only 2.7% of patients with severe DA, making it different from a study by Humphris et al. [15] with almost 10%. One possible explanation could be a reduction in DA levels during periodontal treatment [20]. Furthermore, the sample consisted of only 16% of subjects with no DA, which is an extremely small number and underlines the significance in the clinical setting to counteract DA as well as finding means to treat anxious patients in a suitable way. We can support the findings of Maggiaris et al. [54] and Liau et al. [55] that younger patients tend to have higher DA levels. According to Tickle et al. [18], DA patients seem to find dental treatment more painful, but our results did not imply a significant correlation between the MDAS and HR/EDR. This differs from other findings that demonstrated higher HR rates in patients with higher DA levels [56, 57]. We also found that DA aligns with higher chances of poor OHRQoL [25–27] as well as PTSD and personality disorders. Our study also concludes that the periodontitis classification is related to OHRQoL [58, 59], as we found that a higher stage, grade, and/or extent often also have a poor OHRQoL.

Several limitations must be acknowledged. First, no standardized baseline or resting condition was implemented, so anticipatory arousal during the initial phase may have influenced HR. Second, the use of two different wrist-worn devices introduced heterogeneity; since raw data access was restricted, advanced artifact correction was not possible. In addition, the extremely wide variability of EDR measurements (SD ≈ 150–172 µS) indicates substantial signal dispersion, most likely arising from uncontrolled motion artifacts and the aggregation of tonic and phasic components within the proprietary device output. Because as for this study we chose usability in the clinical setting over experimental control, we did not have access to raw EDR data, or the ability to apply signal transformations or artifact rejection, normalization (e.g., log or square-root transformation) and outlier removal were not feasible. Therefore, EDR data were interpreted descriptively, focusing on within-subject trends rather than absolute values.

A further limitation concerns the statistical approach. We used non-parametric tests (Kruskal–Wallis and Mann–Whitney) for comparisons across intervention clusters, which do not account for the repeated-measures structure and may increase type I error risk. Although mixed-effects models would be methodologically preferable, they were not feasible due to the limited sample size and model instability. Consequently, our analyses emphasize descriptive trends and effect-size estimates, and results should be interpreted with caution.

Although the type of device may have influenced recorded HR values, exploratory analyses did not reveal systematic bias related to device assignment. As each participant was measured with only one device and analyses focused on within-subject trends rather than between-device comparisons, device type was not included as a statistical factor. This limitation has been acknowledged. Finally, the sample comprised multimorbid and medicated patients, including individuals on cardioactive drugs, which may have altered physiological readings. The small sample size limited statistical power and model stability, restricting inference to exploratory interpretation. Due to the clinical nature of the study setting, many participants presented with multimorbidity and long-term medication use. While this introduces variability, it reflects the real-world SPT population and increases the external validity of the findings. Local anesthesia containing epinephrine was used in a small subset of patients (*n* = 4), administered prior to clinical procedures. While this may transiently affect heart rate, the limited number and timing suggest minimal impact on overall findings. As a pilot study, the sample size was not intended to support confirmatory statistical modeling but rather to generate feasibility data and inform the design of future controlled investigations. A summary of methodological limitations and recommendations for future studies is provided in Supplementary Table S2.

Although we conducted an initial sample size calculation and found 51 subjects to be sufficient, our sample size of 75 patients was not large enough considering subgroup analysis, especially as data losses also occurred. As a pilot study, the sample size was not intended to support confirmatory statistical modeling but to inform future research by identifying trends, estimating preliminary effect sizes, and evaluating methodological feasibility in real-world periodontal care. It was not possible to indicate stress levels, as we found no significant differences of HR and EDR throughout the intervention clusters. Even though HR differed between procedural clusters, these fluctuations are more likely attributable to physical activity, patient positioning, or anticipatory tension rather than to psychological stress alone. Accordingly, our interpretation emphasizes physiological variability within the clinical workflow rather than emotional stress per se. There are multiple possible reasons for this. Overall, the stress level during SPT could have been too minor to be measured, especially because SPT patients have already been accustomed to periodontal treatment and know what to expect. It would have been relevant to create simulated settings of stress and of calmness before SPT to indicate the individual HR and EDR baseline and peak rates, thereby being able to compare and rank the measurements during SPT with these two extremes. Another major factor was the use of two different measuring devices, device1 and device2, that did not provide comparable data for HR and EDR. This study’s internal validity is limited by a multimorbid and partially medicated sample, as there was a significant influence of long-term medication and general diseases on HR and EDR. These two factors could have an effect on heart activity, especially cardiovascular diseases and drugs influencing the heart function, which then leads to invalid HR measures with false interpretations of the stress caused by certain interventions. Therefore, cardiovascular diseases and heart medication should have been assessed in greater detail and the respective subjects should have been excluded from the study. In our data, we observed that the interventions at the beginning of the SPT session (e.g., welcome interview) produced higher HR. The subjects could have been more nervous in terms of tensed anticipation, but they calmed down eventually. Otherwise, external factors could also have accelerated the patients’ HR, such as climbing the stairs to the second floor of the periodontology department or being late, which can cause people to be more nervous. Therefore, it is important that a rest period be taken before vital signs are measured in SPT.

## Conclusion

Within the limitations of this observational cross-sectional pilot study, socio-demographic and treatment-related factors appeared intertwined with clinical and psychological parameters influencing adherence to SPT. While HR varied between procedural clusters, no clear link between HR/EDR and psychological stress could be established due to confounding influences. These findings highlight methodological considerations for future, larger, and technically standardized studies.

## Supplementary Information

Supplementary Material 1.

Supplementary Material 2.

## Data Availability

Due to national data-protection regulations, individual-level raw HR/EDR time series cannot be made publicly available. However, anonymized clustered datasets and selected analysis scripts can be shared upon reasonable request to the corresponding author in accordance with ethical approval and institutional agreements.

## Abbreviations

APT: Active periodontal therapy
BOP: Bleeding on probing
CAL: Clinical attachment loss
device1: CARDIOWATCH 287–2 (Corsano Health B.V., AN The Hague, The Netherlands)
device2: Empatica E4 wristband (Empatica Inc., Cambridge MA, United States)
CTS: Childhood Trauma Screener
DA: Dental anxiety
EDR: Electrodermal response
FI: Furcation involvement
GBA: Gemeinsamer Bundesausschuss
GAD: 2–Generalized Anxiety Disorder scale 2
GR: Gingival recession
HR: Heart rate
iC: Invasive intervention cluster
IES: 6–short version of Impact of Event Scale–revised
miC: minimal invasive intervention cluster
MDAS: Modified Dental Anxiety Scale
niC: non-invasive intervention cluster
NSPT: Non–surgical therapy
OHIP: 14–Oral Health Impact Profile
OHRQoL: Oral health–related quality of life
OPD: SQS12–short version of Operationalized Psychodynamic Diagnostic–Structure Questionnaire
PCR: Plaque control record
PD: Probing depths
PHQ: 2–Patient Health Questionnaire 2
PHQ: 4–Patient Health Questionnaire 4
PTSD: Post–traumatic stress disorder
SPT: Supportive periodontal therapy
TM: Tooth mobility
