# Supplementary material for: Supportive periodontal therapy: individual patients’ perception of various professional interventions
Source: BMC Oral Health. 2026 Jan 16;26:235. doi: 10.1186/s12903-026-07656-5 (PMC12870894; doi:10.1186/s12903-026-07656-5)
Supplement: Supplementary file 1 — Supplementary Material 1. [file 12903_2026_7656_MOESM1_ESM.pdf]

Table S1. Overview of all fourteen recorded clinical interventions during SPT sessions and their assignment to one of three intervention clusters (non-invasive (n-iC), minimally invasive (m-iC), invasive (iC)) based on the expected degree of physical manipulation and psychophysiological activation. Cluster definitions reflect a pragmatic, clinical rationale as outlined in the main text and were used to group interventions for statistical analysis.

No.	Intervention	Assigned Cluster	Rationale for Classification
1	Welcome interview	Non-invasive (n-iC)	No physical contact; verbal communication only
2	Extraoral/intraoral examination	Non-invasive (n-iC)	Visual/manual inspection without instrumentation
3	Measurement of periodontal parameters (PD, GR, BOP, TM, FI)	Minimally invasive (m-iC)	Light probing with potential for mild discomfort
4	Local anesthesia	Invasive (iC)	Injection involving tissue penetration
5	Instrumentation with curettes	Invasive (iC)	Mechanical subgingival debridement
6	Instrumentation with sonic scaler	Invasive (iC)	Mechanical instrumentation with vibrational effect
7	Instrumentation with ultrasonic scaler	Invasive (iC)	High-frequency instrumentation, potentially uncomfortable
8	Instrumentation with airflow	Minimally invasive (m-iC)	Supragingival application with moderate physical sensation
9	Rubber cup with polishing paste	Minimally invasive (m-iC)	Surface contact, generally well tolerated
10	Use of oral hygiene aids (e.g., toothbrush, floss)	Minimally invasive (m-iC)	Mild manipulation, no tissue trauma
11	Interdental rotating brushes	Minimally invasive (m-iC)	Slight mechanical stimulation
12	Fluoridation/subgingival medication application (e.g., chlorhexidine gel)	Non-invasive (n-iC)	Passive application, no active instrumentation or scraping
13	Patient-clinician interaction (oral hygiene instruction, Q&A)	Non-invasive (n-iC)	Verbal communication only
14	Treatment breaks (e.g., patient waits for dentist)	Non-invasive (n-iC)	No clinical activity, passive waiting phase
