# Supplementary material for: Supportive periodontal therapy: individual patients’ perception of various professional interventions
Source: BMC Oral Health. 2026 Jan 16;26:235. doi: 10.1186/s12903-026-07656-5 (PMC12870894; doi:10.1186/s12903-026-07656-5)
Supplement: Supplementary file 2 — Supplementary Material 2. [file 12903_2026_7656_MOESM2_ESM.pdf]

The following table S2 summarizes key methodological limitations encountered in this pilot study, along with recommended strategies for future research aiming to measure psychophysiological stress markers in clinical dental settings.

Table S2: Methodological limitations and considerations for future studies

Aspect	Current study limitation	Recommendation for future research
**Signal preprocessing (HR/EDA)**	Raw time series not available; limited insight into device-internal filtering and artifact handling	Ensure access to raw physiological data; apply custom filters and motion artifact rejection pipeline
**Statistical modeling**	Non-parametric tests used due to small sample and pilot design; no mixed-effects models applied	Use linear mixed-effects models including subject/device as factors and adjust for covariates and clustering
**Device variability**	Two different wearable devices used (one per patient); no within-subject device comparison possible	Standardize measurement devices; validate agreement if multiple devices are required
**Measurement context**	No standardized pre-measurement rest, no control of posture, caffeine, nicotine, etc.	Define a pre-measurement rest protocol; standardize measurement time and environmental conditions
**Local anesthesia**	Used in 4 patients only; administered before measurements started	Either exclude such cases or model anesthesia timing as a covariate if frequent
**Intervention-level granularity**	Interventions analyzed as clusters (non/minimally/invasive)	Consider single-intervention level analysis for finer resolution, where sample size allows
**Effect estimates and reporting**	Exact p-values and CIs provided for key outcomes only; some thresholds used for clarity	Report exact p-values, 95% CIs, and standardized effect sizes for all key contrasts
**Visualizations**	Standard plots used; EMMeans and spaghetti plots not included	Include within-subject trajectories, cluster-level EMMeans ± CI plots, and agreement plots when relevant
**Data/code availability**	Raw physiological data restricted due to institutional policy	Share anonymized summary data and analysis scripts where possible; clarify data access upon request
